# Early predictors in language-based learning disabilities: a bibliometric analysis

**DOI:** 10.3389/fpsyt.2023.1229580

**Published:** 2023-12-04

**Authors:** Maryam Alabbad, Muhammad Ajmal Khan, Nadeem Siddique, Jaber Abou Hassan, Shahid Bashir, Turki Abualait

**Affiliations:** ^1^Department of Medical Rehabilitation and Long-Term Care, Al-Ahsa Health Cluster, Al-Ahsa, Saudi Arabia; ^2^College of Applied Medical Sciences, Imam Abdulrahman Bin Faisal University, Dammam, Saudi Arabia; ^3^Deanship of Library Affairs, Imam Abdulrahman Bin Faisal University, Dammam, Saudi Arabia; ^4^Library Department, Lahore University of Management Sciences, Lahore, Pakistan; ^5^Neuroscience Center, King Fahad Specialist Hospital, Dammam, Saudi Arabia

**Keywords:** early predictors, dyslexia, dyscalculia, dysgraphia, modalities

## Abstract

**Introduction:**

Language-based learning disabilities (LBLD) refers to a spectrum of neurodevelopmental-associated disorders that are characterized by cognitive and behavioral differences in comprehending, processing and utilizing spoken and/or written language. The focus of this work was on identifying early predictors of three main specific LBLD including dyslexia, dyscalculia, and dysgraphia.

**Methods:**

The Web of Science (WoS) was searched for literature related to (neurocognitive, neurophysiological, and neuroimaging) measurements used to identify early predictors of LBLD from 1991 to 25 October 2021. A retrospective bibliometric analysis was performed to analyze collaboration among countries, institutions, authors, publishing journals, reference co-citation patterns, keyword co-occurrence, keyword clustering, and burst keywords using Biblioanalytics software.

**Results:**

In total, 921 publications related to the identification of LBLD using (neurocognitive, neurophysiological, and neuroimaging) modalities were included. The data analysis shows a slow growth in research on the topic in the 90s and early 2000 and growing trend in recent years. The most prolific and cited journal is Neuroimage, followed by Neuropsychologia. The United States and Finland’s Universities Jyvaskyla and Helsinki are the leading country and institution in this field, respectively. “Neuroimaging,” “brain,” “fMRI,” “cognitive predictor,” “comorbidity,” “cortical thickness” were identified as hotspots and trends of (neurocognitive, neurophysiological, and neuroimaging) modalities in the identification of LBLD.

**Discussion:**

Early predictors of LBLDs would be useful as targets for specific prevention and intervention programs to be implemented at very young ages, which could have a significant clinical impact. A novel finding of neuroimaging predictors combined with neurocognitive and neuropsychological batteries may have implications for future research.

## 1 Introduction

Language-based learning disabilities (LBLD) refers to a spectrum of neurodevelopmental-associated disorders that are characterized by cognitive and behavioral differences in comprehending, processing and utilizing spoken and/or written language (1). LBLD can manifest as a wide array of language difficulties or impairments, including experience troubles with speaking, reading, spelling, writing, math, and listening, with different levels of severity (2). LBLD is a multifactorial disability which can result from a combination of developmentally neurobiological variability changes in the brain function and environmental factors, in absence of neurological, psychiatric or mental disorders, and genetic syndromes (3). Various reports state the frequency and prevalence of LBLDs, with varying rates depending on sample size and inclusion criteria. For example, Al-Yagon et al. (4) reported varying prevalence rates, including 1.2% from a Greek epidemiologic study in 2004 and 20.0% from an Australian study in 2000. In the United States, the 2003 National Survey of Children’s Health (NSCH) reported a lifelong prevalence estimate of learning disability of 9.7% in children aged 3 to 17 years old (5). In Turkey, the probable prevalence rate was found to be 13.6% in a checklist-based epidemiological study of 2,174 primary school children (6). LBLDs is two to three times more common in boys than in girls, according to the DSM-5 (4).

LBLD are typically emerge in children during the early years of education however, some children may exhibit significant learning difficulties later, which indicates that diagnosis of such disabilities might be made at any time after formal education begins including adolescence and adulthood (7). LBLD is a common cause of academic incompetence as difficulties in language and literacy skills hinder comprehension and communication capabilities (8). Evidence has shown that early assessments of children’s language skills, at preschool level, predict their future learning and academic performance, indicating that language acquisition trajectories are influenced by preschool experiences (2). Furthermore, many studies have shown that LBLDs have significant impact on movement/physical disabilities and neurodevelopmental milestone, indicating inefficient nervous system development and/or exhibit delayed fine motor or gross motor control during childhood (9).

The term LBLD is an umbrella term comprising a wide range of diverse deficits with learning particularly in reading, writing, math, and problem solving (2). Other disorders such as attention deficit hyperactivity disorder (ADHD) or autism spectrum disorder (ASD) also affect learning skills but they are not considered to be learning disabilities (10, 11). ADHD or ASD impacts more global skills and cognitive/executive functions compared to LBLD (12, 13). Thus, some challenging effects of the ADHD or ASD, such as difficulty staying focused and paying attention, hyperactivity, behavioral, interaction and communication impairments may impact individual’s ability to learn (13, 14). Although such disorders might co-exist with LBLD and share some common manifestations or characteristics they are distinct from each other and have discrete underlying neural basis (15). Therefore, the focus of this work was on three main specific language-based learning developmental disorders, apart from comorbidities, including dyslexia, dyscalculia, and dysgraphia. Developmental dyslexia is defined as inability to read characterized by chronic difficulty in decoding, fluency, comprehension and word recognition (16). Developmental dyscalculia hinders the development of mathematical reasoning and skills (17). Developmental dysgraphia is a condition that manifests as difficulty acquiring writing and spelling, punctuation and handwriting competencies despite proper education, vision, and intelligence quotient (18). These aforementioned developmental disorders can be observed individually or in groups (7). Behavioral, neurocognitive, neurophysiological and neuroimaging measures would be useful in elucidating the underlying process of language and learning disorders which would be crucial for identifying early predictors of such disorders. Therefore, a diagnosis and proactive approaches of evaluations to identify early predictors of LBLD would be helpful as targets for specific prevention and/or intervention programs to be applied at very young ages.

Bibliometric analysis method has been extensively applied in scientific research studies (19). Bibliometric analysis starts revealing the aspect that still possesses many of the enduring enquiries (20). Therefore, a better understanding of the most important advancements achieved in the LBLD research field over the last few decades can be obtained by analyzing the most referenced papers. Several developmental LBLDs have been studied using the bibliometric approach, for example dyslexia (21). Nonetheless, to the best of our knowledge, no bibliometric analyses have been carried out in the field of LBLDs such as dyslexia, dysgraphia, and dyscalculia combined all together. Hence, the aim of the current study was to analyze the top-cited studies related to early neurocognitive, neurophysiological and neuroimaging predictors of LBLDs centering on dyslexia, dysgraphia, and dyscalculia, collectively. In the current study, we used bibliometrics and literature visualization tools to examine the global research status of early predictors of LBLD from 1991 to 25 October 2021. The findings are presented in the form of a visual map to help researchers better understand the research hotspots, future trends, and application prospects of early predictors of LBLD.

## 2 Methods

### 2.1 Search strategy

Research on neuro-markers for learning-based language disorders was analyzed using bibliometric techniques, which were taken advantage of in this study. Data from the Web of Science Core Collection, one of the most comprehensive and trustable data sources, was used in the analysis. The researchers conducted an exhaustive literature retrieval on 20 May 2021, regardless of publication year, country of origin, or language. The researchers rigorously identified all possible relevant keywords for the retrieval of all related publications. On 25 October 2021, the following query was entered into the main search field of the Web of Science database:

TS = (Dyslexia OR Dyscalculia OR Dysgraphia OR “reading disorder” OR “spelling disorder” OR “reading disability” OR “spelling disability” OR “reading difficulty” OR “writing disorder” OR “writing disability” OR “writing difficulty” OR “arithmetical difficulty”) AND TS = (“Brain imaging” OR “Neurocognitive” OR “Neuroimaging” OR “Cognitive biomarker*” OR “Cognitive predictor*” OR “late discriminative negativity” OR “Mismatch Negativity” OR “Mismatch Response”).

The query resulted in 962, the document types of Editorial Materials (21), Meeting Abstracts (12), Early Access (8), Letters (2), News Items (2), Biographical-Items (1), Corrections (1), Notes (1), and Retracted Publications (1) were excluded from the research. As a result, a total of 921 documents that included Article (731), Book Chapter (14), Proceeding Paper (23), and Review (153) documents were downloaded in CSV, RIS, and BIB formats. The data were imported into EndNote, a citation management software, to perform a duplicate check on the author, title, and year. There were no duplicate records found in the data. Out of 921 documents, there were 250 documents with no author-supplied keywords. Therefore, the researchers prepared a list of author-supplied keywords from the available author-supplied keywords (in 921–250 = 671 documents) with a frequency of greater than 10. The title and abstract are also provided by the authors. Hence, a new field by combining the three fields (title, abstract, and author keywords) was created. The list of these author-supplied keywords was searched by using the Biblioanalytics software in the new field created by combining the above-mentioned fields (containing the data of all 921 documents). The Biblioanalytics software provides such a facility and the retrieved results were noted down. In this way, the author-supplied keywords from all 921 documents were extracted. A criterion was set with a frequency of over 10 articles to only include author-supplied keywords. The country and organization of the authors were confirmed with the help of C1 (Affiliation) and RP (corresponding author address). The study used Biblioshiny, Power BI, MS Access, MS Excel, Biblioanalytics, and an online visualization platform^1^ for data analysis.

### 2.2 Statistical analysis

The frequency was calculated using SPSS 11.0 (Chicago, IL, United States). We looked at the following information: the number of citations, the year of publication, the country, the first author, the journal, the language, the type of study, and the Web of Science subject category. Two authors independently searched the abstracts and full texts to determine which LBLD related articles received the most citations. Through discussion, the authors were able to work out their differences. Only research that specifically addressed the predictors of developmental (dyslexia, dyscalculia, and dysgraphia) was considered for inclusion in the subsequent analyses. The authors did not consider those studies that only made a passing reference to LBLD. Total article citation counts were used to compile the final list of LBLD developmental studies (dyslexia, dysgraphia, and dyscalculia). For each article, the title, authors, journal, language, total number of citations, publication year, nation, journal impact factor, article type, and Web of Science subject category were extracted. If the reprint author had two or more affiliations from different countries, we used the first affiliation as the country of origin. The first category was chosen if an article appeared in more than one subject category. Table 1 presents the highly cited articles related to different measures that identify early predictors of LBLD. In the case of multiple authors, we have only shown the first author of the articles just for reference purposes.

**Table 1:** Highly cited articles related to different measures that identify early predictors of LBLD.

Paper	Total citations	TC per year
MCCANDLISS BD, 2003, TRENDS COGN SCI	980	51.5789
ULLMAN MT, 2004, COGNITION	855	47.5
STEIN J, 1997, TRENDS NEUROSCI	741	29.64
PAULESU E, 2001, SCIENCE	679	32.3333
FIEZ JA, 1998, P NATL ACAD SCI USA	547	22.7917
DEHAENE S, 1998, TRENDS NEUROSCI	535	22.2917
BENTIN S, 1999, J COGNITIVE NEUROSCI	521	22.6522
GRODZINSKY Y, 2000, BEHAV BRAIN SCI	516	23.4545
TEMPLE E, 2003, P NATL ACAD SCI USA	501	26.3684
EDEN GF, 1996, NATURE	485	18.6538
SHAYWITZ SE, 2005, BIOL PSYCHIAT	480	28.2353
TURKELTAUB PE, 2003, NAT NEUROSCI	462	24.3158
RAYMOND AA, 1995, BRAIN	455	16.8519
SEIDMAN LJ, 2005, BIOL PSYCHIAT	446	26.2353
DEHAENE S, 2004, CURR OPIN NEUROBIOL	434	24.1111
PAULESU E, 1996, BRAIN	417	16.0385
TANNOCK R, 1998, J CHILD PSYCHOL PSYC	415	17.2917
HABIB M, 2000, BRAIN	402	18.2727
PUGH KR, 2000, MENT RETARD DEV D R	375	17.0455
NORTON ES, 2012, ANNU REV PSYCHOL	361	36.1

## 3 Results

### 3.1 Analysis of quantity and annual trend of published literature

Table 2 illustrates the yearly productivity of predictors for developmental disorders research. The data indicate a slow growth in the research on the topic with only single-digit publications from 1991 to 1997 with a gap of 2 years as no publication appeared in 1992 and 1993. After 1997, almost a consistent growth with a little variation has been observed, with 2018 as the top year contributing the highest number of publications followed by 2015, 2016, and 2020. According to the citation analysis, 2003 was the year with the highest number of citations, followed by 2006 and 2005. The further analysis regarding U1 (Usage Count in Last 180 Days) placed 2021 at the first position, followed by 2018 and 2020. Likewise, the analysis regarding U2 (Usage Count Since 2013) placed 2015 at the top position, followed by 2013 and 2012.

**Table 2:** Yearly productivity related to different measures that identify early predictors of LBLD.

Years	TP	TC	U1	U2
1991	2	27	0	1
1994	6	304	0	28
1995	4	586	0	38
1996	6	1,119	3	134
1997	7	888	3	157
1998	19	2,553	4	315
1999	19	1,696	5	262
2000	16	2,129	3	505
2001	17	1,642	6	309
2002	22	1,175	5	208
2003	21	3,128	14	486
2004	22	2,517	23	737
2005	31	2,855	17	792
2006	46	2,883	9	774
2007	37	2,707	19	814
2008	45	2,761	20	1,063
2009	44	2,808	27	970
2010	31	1,662	42	861
2011	43	2,070	75	1,236
2012	42	2,674	91	1,633
2013	43	1,934	55	1,719
2014	38	1,046	67	1,262
2015	54	1,353	123	,1952
2016	54	927	122	1,476
2017	42	585	100	798
2018	64	589	187	1,101
2019	48	336	136	749
2020	51	161	179	486
2021	47	39	201	279
Grand total	921	45,154	1,536	21,145

U1, Usage Count (Last 180 Days); U2, Usage Count (Since 2013).

### 3.2 Document type analysis

921 literature types were screened. Article was the most frequently published category of literature, accounting for (731, 79.37%) of the total literature. Review was the second largest category of literature type (153, 16.61%). Finally, Book Chapter (14, 1.52%), and Proceeding Paper (23, 2.49%) were the third and fourth largest literature type. The citation analysis also ranked “Article” at the first position, distantly followed by “Review.” Whereas the document type “Book Chapter” maintained the third position in securing citations. The analysis regarding U1 (Usage Count in Last 180 Days) and U2 (Usage Count Since 2013) also placed “Article” (Table 3).

**Table 3:** The document types preferred by the researchers related to different measures that identify early predictors of LBLD.

Document type	TP	TC	U1	U2
Article	731	29,497	1,190	14,339
Book chapter	14	1,266	84	918
Proceedings paper	23	71	4	69
Review	153	14,320	258	5,819
Grand total	921	45,154	1,536	21,145

### 3.3 Authorship pattern

Table 4 depicts the authorship patterns in the topic of the study ranging from one author to 49 author patterns. In the analysis, the three-author pattern emerged as the most preferred pattern with the highest number of publications, followed by four and two-authored patterns. The citation-wise analysis also ranked the three-author pattern at the top due to securing the highest number of citations, followed by two and single-author patterns. The further analysis regarding U1 (Usage Count in Last 180 Days) placed the two-author pattern at the first position, followed by three and five-author patterns. Likewise, the analysis regarding U2 (Usage Count Since 2013) placed the two-author pattern at the top position, followed by three and single-author patterns.

**Table 4:** Authorship pattern related to different measures that identify early predictors of LBLD.

Author(s)	TP	TC	U1	U2
1	94	5,832	104	2,807
2	134	7,468	276	3,951
3	153	8,195	234	3,795
4	138	4,821	219	2,558
5	121	5,278	228	2,477
6	89	3,894	125	1,626
7	55	2,946	79	1,069
8	43	2,140	71	795
9	26	919	53	502
10	17	714	31	423
11	16	1,327	30	457
12	7	323	18	127
13	6	171	19	86
14	5	120	14	119
15	4	583	2	76
16	2	15	4	13
17	1	3	0	9
19	2	11	5	7
21	1	193	4	109
26	2	17	4	29
30	1	63	2	19
31	1	37	4	14
41	1	41	2	43
45	1	31	3	22
49	1	12	5	12
Grand total	921	45,154	1,536	21,145

### 3.4 Most productive authors with impact

The most prolific researchers related to different measures that identify early predictors of LBLD with their impact have been portrayed in Table 5. The researcher “LYYTINEN H” emerged as the leading author contributing the highest number of publications on the topic, followed by “KUJALA T,” “LEPPANEN PHT,” and “FLETCHER JM.” The researcher “LYYTINEN H,” who leads all the researchers in publishing research on the topic, also ranked at the top due to the highest h-index, g-index, and m-index.

**Table 5:** Most productive authors with impact related to different measures that identify early predictors of LBLD.

Element	h_index	g_index	m_index	TC	NP	PY_start
LYYTINEN H	20	23	0.8	1,368	23	1997
KUJALA T	13	20	0.591	882	20	2000
LEPPANEN PHT	16	20	0.64	1,312	20	1997
FLETCHER JM	12	19	0.429	652	19	1994
EDEN GF	14	16	0.538	2,027	16	1996
PUGH KR	10	16	0.435	1,275	16	1999
NAATANEN R	15	15	0.682	1,213	15	2000
SCHULTE-KORNE G	11	15	0.458	692	15	1998
GAAB N	9	14	0.563	633	14	2006
GABRIELI JDE	11	14	0.579	1,680	14	2003
PAPANICOLAOU AC	8	13	0.364	233	13	2000
SIMOS PG	8	13	0.364	233	13	2000
SHAYWITZ BA	9	12	0.321	2,041	12	1994
SHAYWITZ SE	9	12	0.321	2,041	12	1994
HEIM S	8	11	0.444	240	11	2004
LANDI N	6	11	0.429	161	11	2008
MARIEN P	8	11	0.471	527	11	2005
BARTLING J	9	10	0.375	585	10	1998
GUTTORM TK	10	10	0.435	846	10	1999
HABIB M	6	10	0.273	1,219	10	2000

### 3.5 Analysis of contributions of journals

The data in Table 6 highlight the most popular outlets for publishing research on the topic. According to the analysis, the most preferred journals for researchers to share their research are “Neuropsychologia,” “Neuroimage,” and “Frontiers in human neuroscience.” The journal “Neuroimage” emerges as the top journal in terms of citations, closely followed by “Neuropsychologia.” The data also disclose the journals with total cited publications (CP) and not cited publications (NCP). Out of 10 top journals, there are six journals, which have one publication with no citation. Likewise, the data also calculate the impact (TP/TC) of the journals. The journal “Neuroreport” which is at the bottom in terms of publications, stands at the top position in terms of impact, followed by “Cortex,” and “Neuroimage.”

**Table 6:** Most productive journals related to different measures that identify early predictors of LBLD.

Journal	NCP	CP	TP	TC	Impact
Neuropsychologia	1	42	43	1,473	34.25581
Neuroimage	1	35	36	1,920	53.33333
Frontiers in human neuroscience		26	26	399	15.34615
Clinical neurophysiology	1	21	22	920	41.81818
Human brain mapping	1	18	19	988	52
Cortex		18	18	997	55.38889
Brain and language		18	18	495	27.5
Frontiers in psychology	1	16	17	495	29.11765
Journal of learning disabilities	1	13	14	567	40.5
Neuroreport		14	14	1,112	79.42857

### 3.6 Top highly cited articles

Top highly cited articles are displayed in Table 1. The article “MCCANDLISS BD, 2003, TRENDS COGN SCI” secured the top position in the list by obtaining the highest number of citations, followed by “ULLMAN MT, 2004, COGNITION,” “STEIN J, 1997, TRENDS NEUROSCI” and “PAULESU E, 2001, SCIENCE.” The data also present per year total citation by dividing total citations by the difference between the publication year and the current year. The articles “MCCANDLISS BD, 2003, TRENDS COGN SCI,” “ULLMAN MT, 2004, COGNITION,” “NORTON ES, 2012, ANNU REV PSYCHOL,” and “PAULESU E, 2001, SCIENCE” maintained first, second, third, and fourth positions, respectively.

### 3.7 Most productive countries

**Figure 1:**
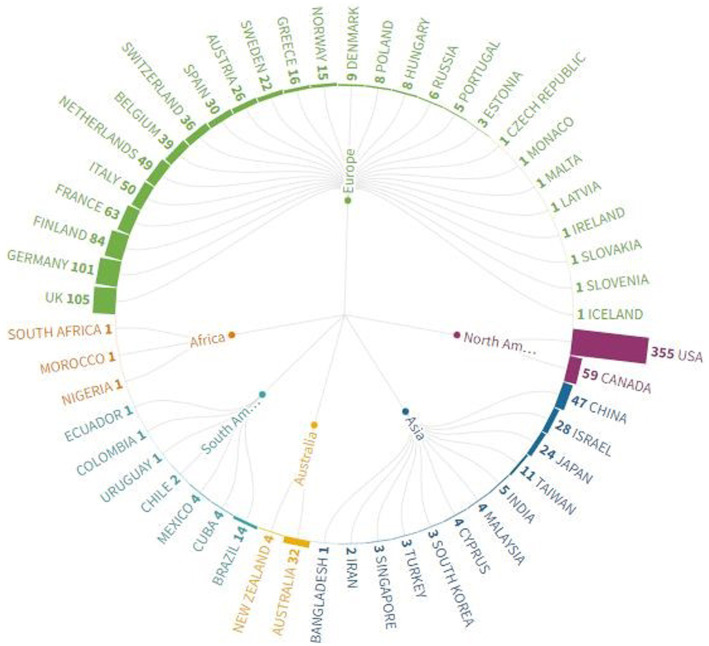
Most productive countries related to different measures that identify early predictors of LBLD.

Figure 1 depicts the research productivity of countries and continents on the topic. The United States emerged as the leading country globally and from North America in terms of the number of publications. The European countries seem as securing the remaining top four positions on the figure. The United Kingdom, Germany, Finland, and France showed a remarkable contribution and maintained second, third, and fourth positions. Canada from North America grabbed the fifth position. China emerged as the leading Asian country with the most publications from the continent, followed by Israel and Japan. Brazil emerged as the top country from South America. Three countries from Africa contributed only three publications, with one publication from each country.

### 3.8 Country production analysis

**Figure 2:**
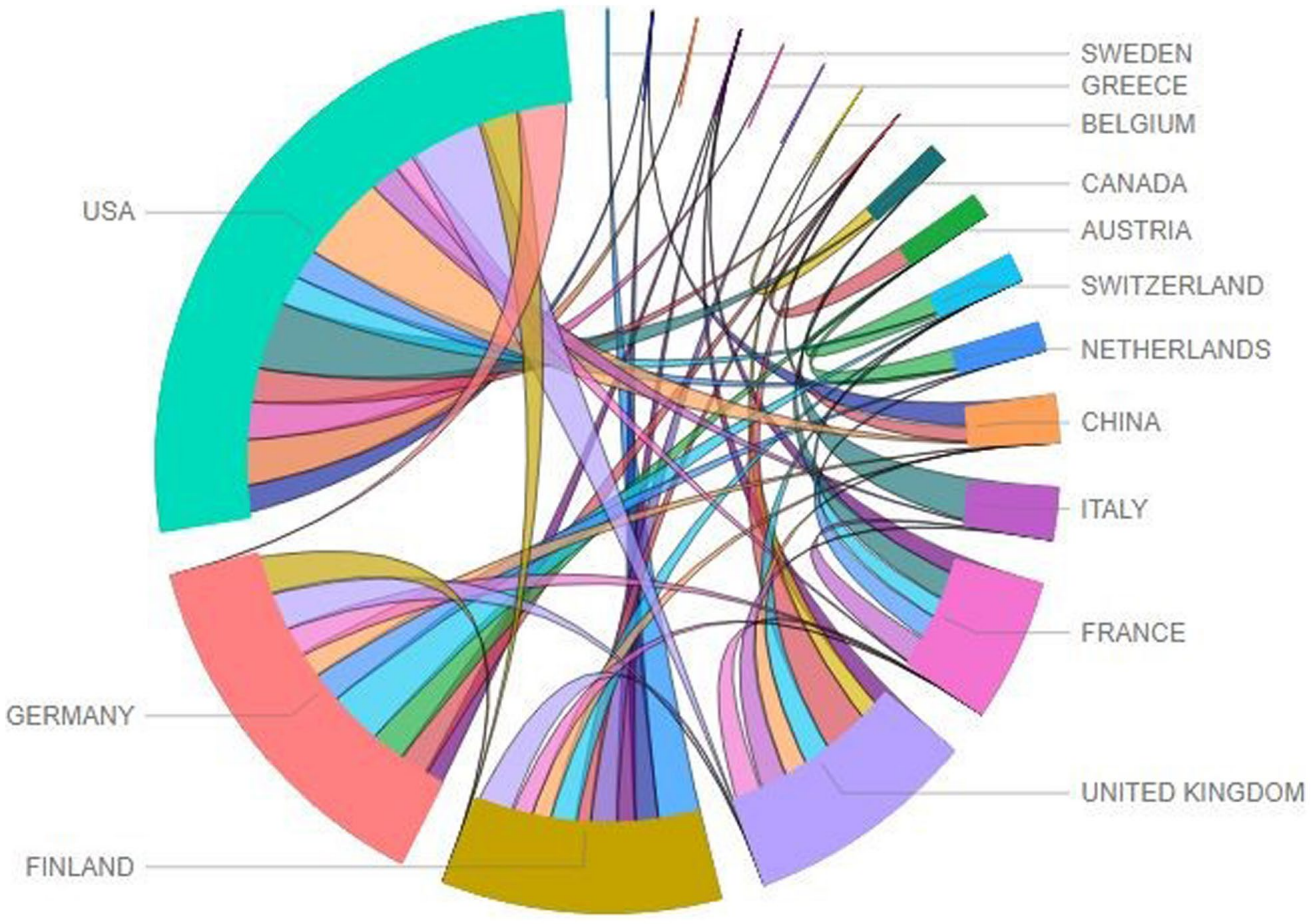
Country collaboration map related to different measures that identify early predictors of LBLD.

Figure 2 illustrates the collaboration among countries in publishing research related to different measures that identify early predictors of LBLD. The United States is the most prolific country in publishing research on the topic, with the highest collaboration with China, followed by the United Kingdom and Canada. The fourth highest collaborative activities can be witnessed on the map between Germany and Switzerland. Other vital collaborations are between the USA and Germany, the United Kingdom and Australia, the USA, and Israel.

### 3.9 Organizations production analysis

**Figure 3:**
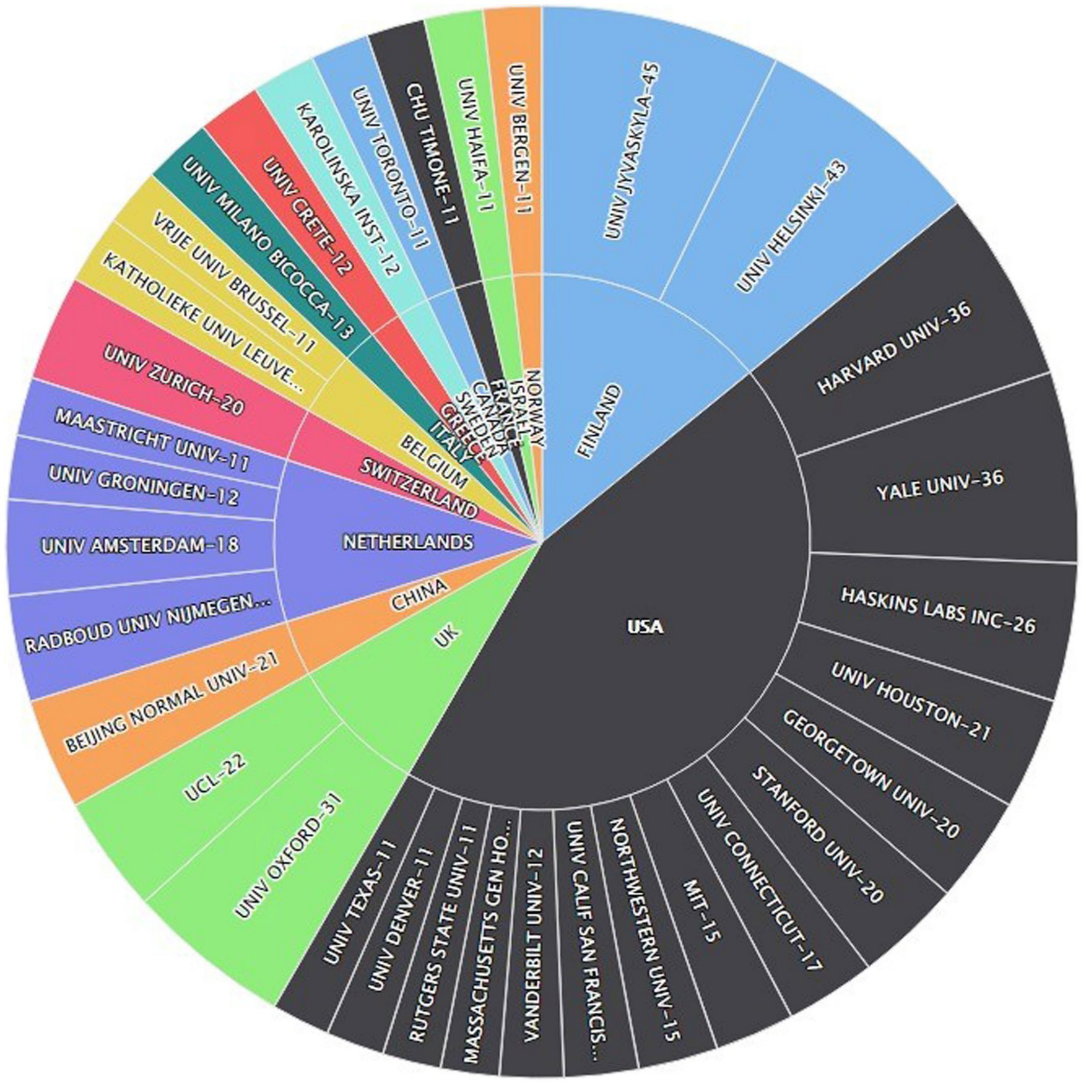
Most productive organizations related to different measures to identify early predictors of LBLD.

Figure 3 portrays highly productive organizations from around the globe in publishing research related to different measures that identify early predictors of LBLD. Two organizations from Finland “University Jyvaskyla” and “University Helsinki” emerged as the most prolific organizations by producing 45 and 43 publications. From the USA, Harvard and Yale Universities contributed 36 publications each and maintained the third position. The University of Oxford appeared as the fourth most productive organization publishing research on the topic. No organization from Germany could appear in the top productive organization graph even though Germany is the third most productive country in the world. Likewise, only one organization from the United Kingdom, France, Canada, and China showed up in the most productive organizations map. However, these countries secured top positions in the most productive countries analysis.

### 3.10 Analysis of author keywords

**Figure 4:**
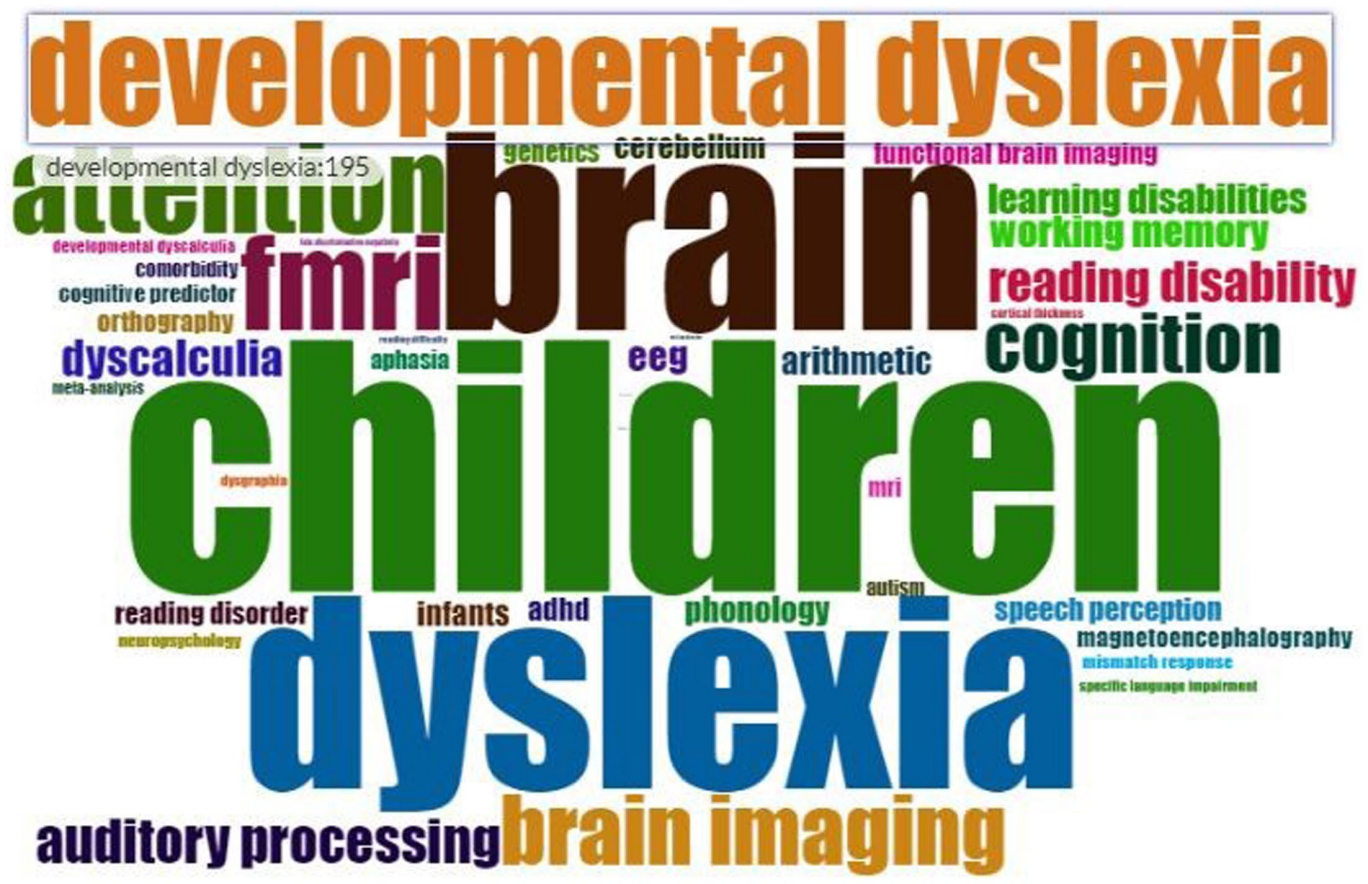
Word cloud of author keywords related to different measures that identify early predictors of LBLD.

During the data analysis, 250 records were found without any author-supplied keywords. Therefore, to fill this gap, prominent keywords were identified from the titles and abstracts of the documents with the help of BiblioAnlytics software. Figure 4 describes the most frequently used keywords related to different measurement modalities that identify early predictors of LBLD. The keyword “children” emerged as the most frequently used keyword. Other prominent frequently used keywords on the graph include “dyslexia,” “brain,” “developmental dyslexia,” “brain imaging,” and “auditory processing.”

### 3.11 Topic trends in research related to different measures that identify early predictors of LBLD

**Figure 5:**
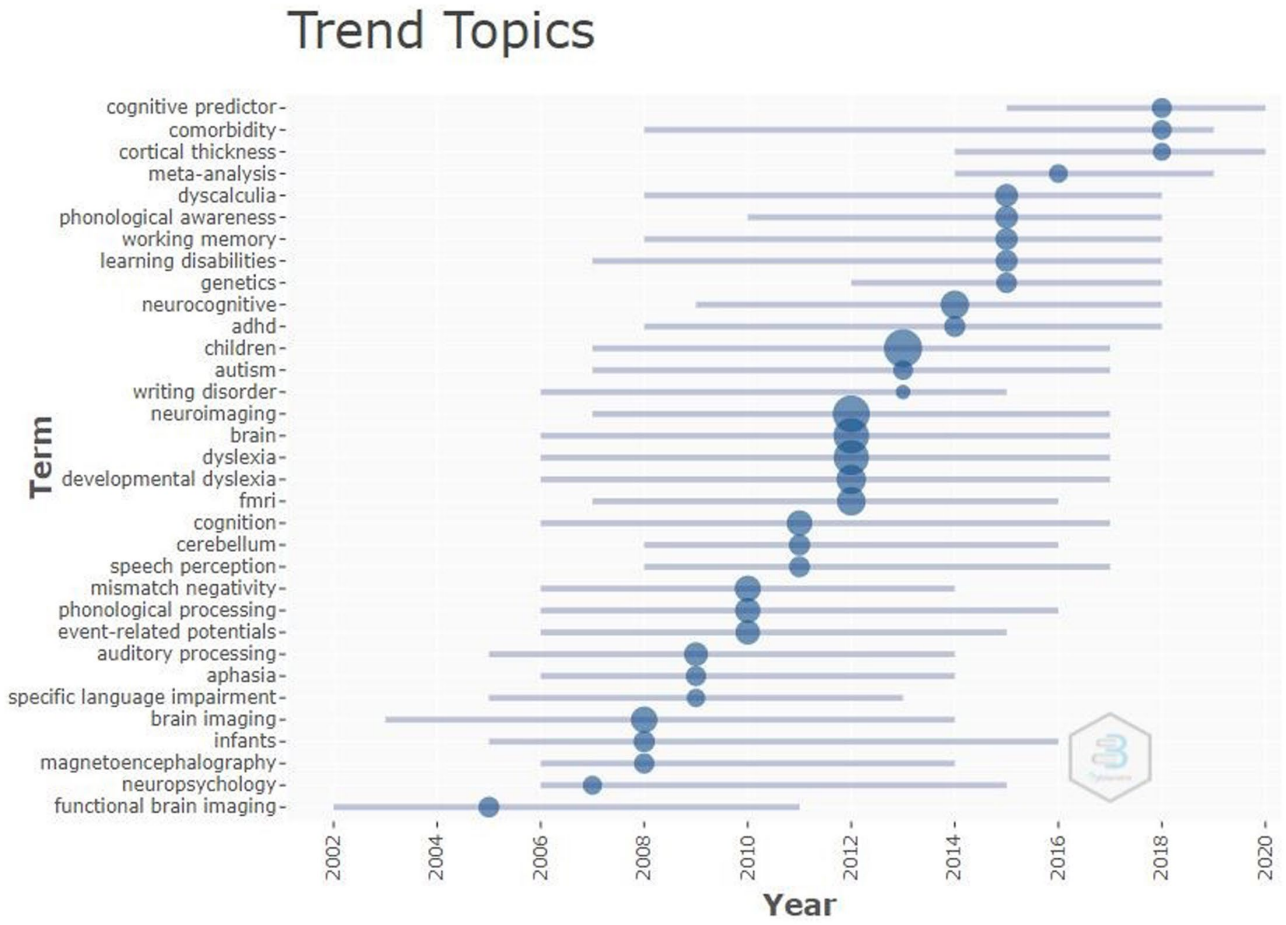
Topic trends related to different measures that identify early predictors of LBLD.

Figure 5 depicts topic trends in the LBDL predictors research. The analysis included the author keywords with a minimum frequency of five and at the least, appeared five times a year. The line represents an author keyword timeline, and the size of the bubble is proportional to the number of documents that used the keyword. The bubble is located at the midpoint of the timeline of the author keyword. The author keyword “children” appeared to be the most frequently used keyword between 2007 and 2017, having its mid-year in 2013. Other commonly used keywords included “neuroimaging,” “brain,” “dyslexia.” “developmental dyslexia,” and “fMRI,” each having mid-year at 2012. The keywords “cognitive predictor,” “comorbidity,” and “cortical thickness” were the most recently used author keywords. However, some keywords like “brain imaging,” “infants,” “magnetoencephalography,” “neuropsychology,” and “functional brain imaging” were not used in the current research.

### 3.12 Analysis of word growth

**Figure 6:**
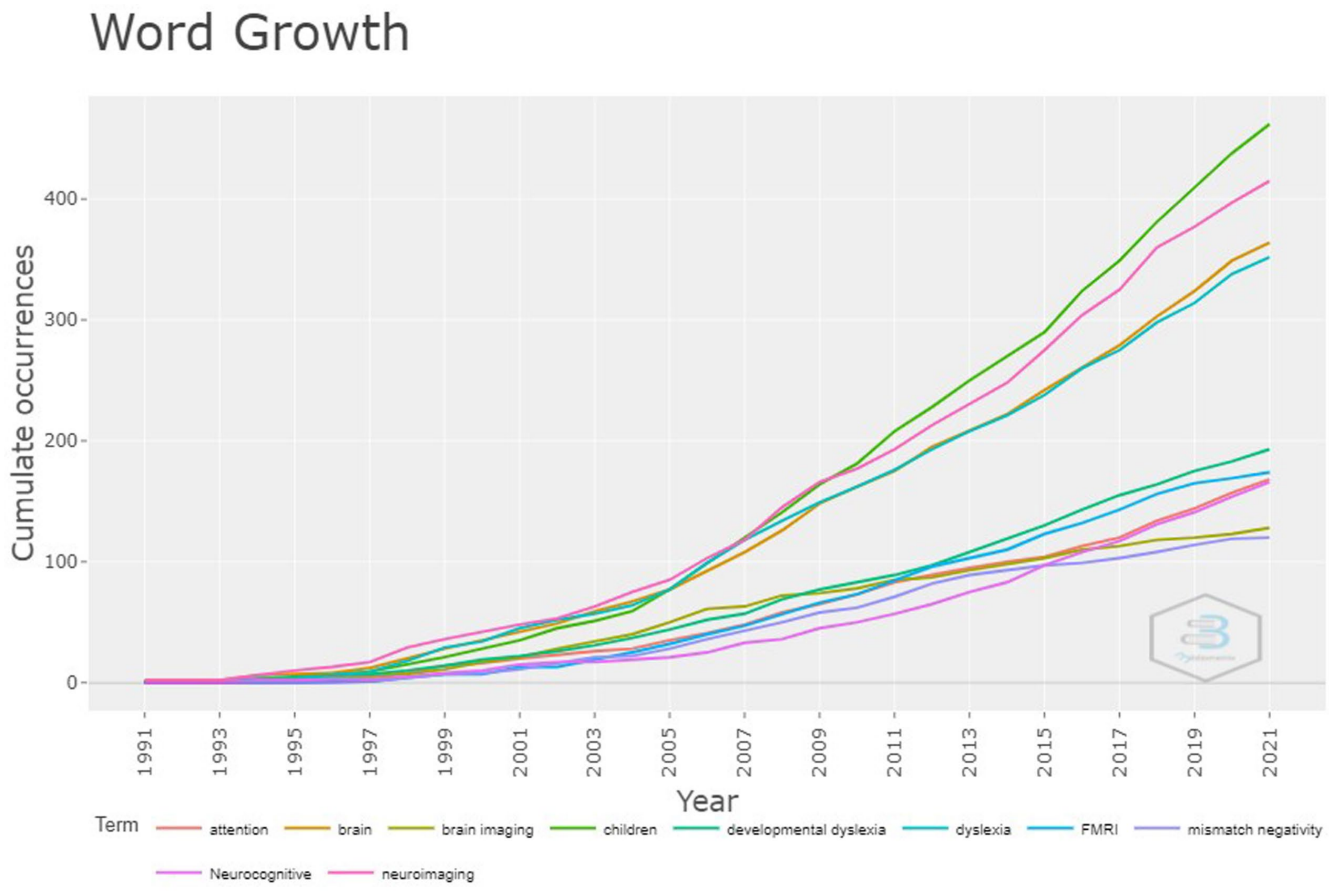
Word growth related to different measures that identify early predictors of LBLD.

Figure 6 illustrates the growth of the author keywords over the years. The keyword “children” was used earlier by authors on the topic and consistently grew over the years. The second most prominent keyword was “neuroimaging,” used by the authors from the beginning to the time of this study with little variation in its usage. The keywords “brain” and “dyslexia” were the other consistently used author keywords.

### 3.13 Analysis of burst author keywords

**Figure 7:**
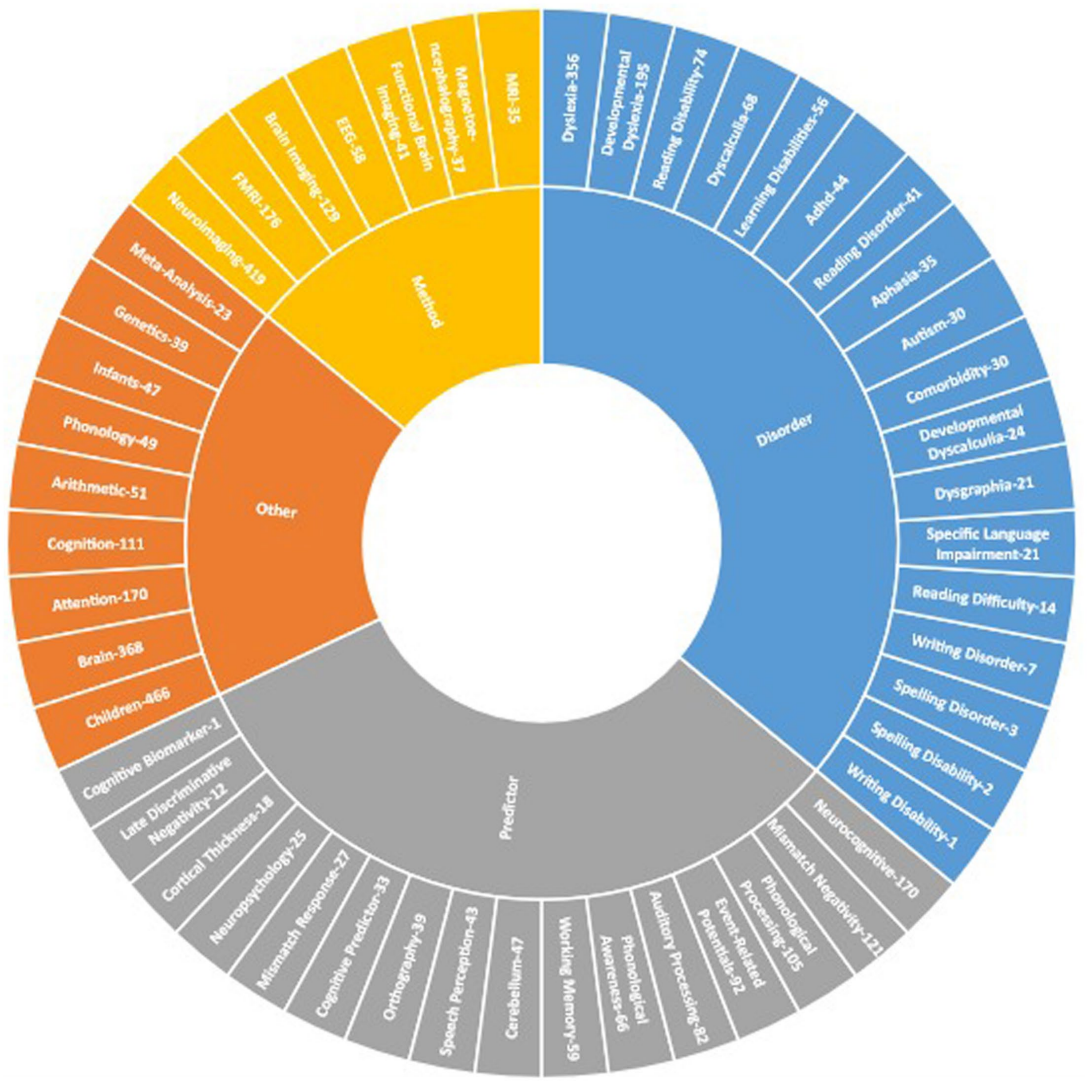
Sunburst of author keywords.

Figure 7 divides the author keywords into four categories, “predictor, “disorder,” “method,” and “other.” The keyword “children” emerged as the most frequently used keyword, followed by “neuroimaging,” “brain,” “dyslexia,” “developmental dyslexia,” and “attention” belonged to the “other,” “method,” “disorder,” and “predictor” categories, respectively.

### 3.14 Analysis of quantity and annual trend of published literature

This study analyzes the correlation between the age of the articles (calculated from the year of publication) and the total citations obtained. Therefore, the normality of the data was tested. Due to the large size of the data, the Kolmogorov–Smirnov test of normality was applied. Since the value of p of both variables was less than 0.05 (*p* = 0.000), it showed that the distributions were not normal. For this reason, a non-parametric test, the Spearman Rank Correlation, was applied to determine the correlation between the two variables Table 7. A significant correlation between the two variables was found (r = 0.538, 0.000). The value of the Spearman Rank Correlation showed a moderate level of correlation between age and total citations obtained.

**Table 7:** Spearman Rank Correlation between age and total citations TC.

Correlations				
			AGE	TC
Spearman’s rho	AGE	Correlation coefficient	1.000	0.538^**^
		Sig. (2-tailed)	.	0.000
		*N*	921	921
	TC	Correlation coefficient	0.538^**^	1.000
		Sig. (2-tailed)	0.000	.
		*N*	921	921

^**^Correlation is significant at the 0.01 level (2-tailed).

### 3.15 Reference analysis

The study further determines the correlation between the number of references (NR) cited and the total citations obtained by the articles. Again, due to the bigger size of the data, the Kolmogorov–Smirnov test of normality was applied to test the normality of the data. Since the value of p of both variables was less than 0.05 (*p* = 0.000), it showed that the distributions were not normal. Hence, a non-parametric test, the Spearman Rank Correlation, was applied Table 8. The analysis showed a significant correlation between the two variables (r = 0.078, 0.018). The value of the Spearman Rank Correlation showed a low level of correlation between the number of references cited and total citations obtained.

**Table 8:** Spearman rank correlation between numbers of references NR cited and total citations TC.

Correlations				
			NR	TC
Spearman’s rho	NR	Correlation coefficient	1.000	0.078^*^
		Sig. (2-tailed)	.	0.018
		*N*	921	921
	TC	Correlation coefficient	0.078^*^	1.000
		Sig. (2-tailed)	0.018	.
		*N*	921	921

^*^Correlation is significant at the 0.05 level (2-tailed).

## 4 Discussion

Several previous bibliometric studies found that research on dyslexia, dyscalculia, and dysgraphia in the past 20 years mainly focused on its etiology (21–26). The current study reviewed the progress of research on multiple measurements modalities including neurocognitive, neurophysiological and neuroimaging for an early evaluation to identify LBLD predictors using Biblioanalytics. In a search of the WoS from 1991 to 25 October 2021, we found 962 cited studies were published in 10 different journals; *Neuropsychologia, Neuroimage, and Frontiers in Human Neuroscience* were the most preferred journals by the researchers for sharing their research. The journal *Neuroimage* emerged as the top journal in securing the highest number of citations, distantly followed by *Neuropsychologia.* Furthermore, the journal *Neuroreport* which was at the bottom in terms of publications, stood in the top position in terms of impact, followed by *Cortex* and *Neuroimage*. The current study on LBLDs will be useful to the research community for the following reasons; first, the current study not only forecasts the future of developmental dyslexia, dyscalculia, and dysgraphia research, but it also identifies associated research trends and gaps in the field. Second, existing findings provide important quantitative information about how both classic studies and recent advances in the field have contributed to a better understanding of LBLD. Third, the current study could help journal editors, funding agencies, and reviewers conduct more in-depth analyses of research articles and grant applications. According to previous findings, a total of 560 contributions related to dyscalculia were published during this period. Most of the articles (92.14%) were written in English as it is the most used language in global publications on dyscalculia. According to the findings, the top 15 authors in the field of dyscalculia wrote 174 (31.07%) of the 560 publications in this field (22). Moreover, during the period from 2015 to 2019, 7,623 authors contributed 1,677 research papers on dyslexia (24). Throughout this quarter, dyslexia-related papers were published. The preferred language of authors is found to be English 1,639 (97.73%) times, according to research on dyslexia.

Indeed, this analytic bibliometric study has the potential to contribute to the literature by adding new information regarding scientific interest in LBLD. However, as with other bibliometric analyses, some limitations must be considered when interpreting the presented findings. First, the search was limited to English-language literature, which makes the analysis incomplete as other language’s literature was not included. Second, biblioanalytics is a professional bibliometric analysis software tool that allows objective analysis arises from the researchers’ perspectives though different researchers may have different perspectives on the same content thus, its intrinsic subjectivity bias is unavoidable.

In conclusion, the etiology of LBLD is complex, and its early detection is crucial in clinical and basic research. Using bibliometric analysis, the trends in the development of different measurements modalities including neurocognitive, neurophysiological and neuroimaging for identifying LBLD predictors can be quantitatively viewed. We identified important publications, authors, journals, institutions, and countries based on 921 articles obtained from WoS, and then analyzed their relationships to reveal the research status of different measurements for the identification of LBLD predictors, as well as hotspots and research fronts. Early predictors of LBLDs would be useful as targets for specific prevention and intervention programs to be implemented at very young ages, which could have a significant clinical impact. A novel finding of neuroimaging predictors combined with neurocognitive and neuropsychological batteries may have implications for future research. In the literature there is abundant of studies about developmental dyslexia and its predictors. However, there is a lack of studies about the developmental dyscalculia and dysgraphia predictors which draws the attention to the importance of conducting research experiments about such disorders given its high prevalence and significant impacts on learning capabilities. Therefore, research on dyscalculia and dysgraphia as learning disabilities is required and would reveal multiple aspects that help in clinical applications by tailoring prevention and therapeutic intervention programs to be implemented at very young ages. Such clinical applications would improve the learning skills and competences that will enhance the academic performance of individuals with learning disabilities. Future research must provide a scientific definition of learning-based language disabilities in developmental dyscalculia and dysgraphia, finalize the neuroimaging, neurophysiological, and neurocognitive predictors, investigate causes and defects, expand research areas, and conduct exhaustive intervention research.

## Data availability statement

The raw data supporting the conclusions of this article will be made available by the authors, without undue reservation.

## Author contributions

TA and SB were responsible for the study ideation, design, and instructions. MS drafted the manuscript. Data collection, analysis and preparing figures and tables were carried out by MS, MK, and NS. JH participated in the revision of the manuscript. All authors contributed to the article and approved the submitted version.

## Footnotes

^1^https://flourish.studio/
